# Ethyl 2-amino-6-benzyl-4,5,6,7-tetra­hydro­thieno[2,3-*c*]pyridine-3-carboxyl­ate

**DOI:** 10.1107/S1600536810051986

**Published:** 2010-12-24

**Authors:** Shuang-Ming Meng, Ke-Wei Wang, Hai Xie, Yue-Qin Fan, Yong Guo

**Affiliations:** a College of Chemistry and Chemical Engineering, Shanxi Datong University, Datong 037009, People’s Republic of China

## Abstract

In the title compound, C_17_H_20_N_2_O_2_S, the tetra­hydro­pyridine ring adopts an envelope conformation with the N atom at the flap position; the phenyl ring makes a dihedral angle of 81.06 (10)° with the thio­phene ring. The amino group links with the carbonyl O atom *via* intra­molecular N—H⋯O hydrogen bonding, forming a six-membered ring. In the crystal, inter­molecular N—H⋯O hydrogen bonds link the mol­ecules into infinite chains running along the *b* axis.

## Related literature

For the biological activity of thio­phene and its derivatives, see: Kidwai & Mishra (2003 ▶); Amr *et al.* (2006 ▶); Sherif (1996 ▶).
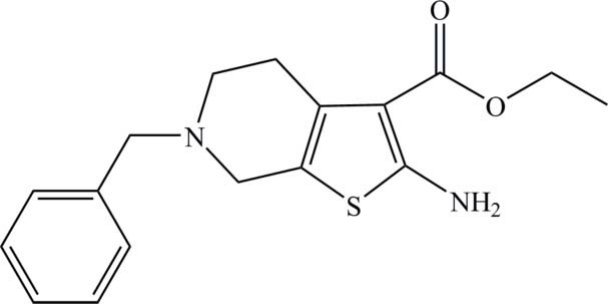


## Experimental

### 

#### Crystal data

C_17_H_20_N_2_O_2_S
*M* *_r_* = 316.41
Monoclinic, 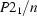

*a* = 12.197 (3) Å
*b* = 9.936 (3) Å
*c* = 13.775 (4) Å
β = 103.430 (4)°
*V* = 1623.8 (8) Å^3^
*Z* = 4
Mo *K*α radiation
μ = 0.21 mm^−1^
*T* = 293 K
0.25 × 0.19 × 0.14 mm

#### Data collection

Bruker SMART APEX CCD diffractometer
Absorption correction: multi-scan (*SADABS*; Bruker, 1999 ▶) *T*_min_ = 0.953, *T*_max_ = 0.977
8867 measured reflections
2875 independent reflections
2122 reflections with *I* > 2σ(*I*)
*R*_int_ = 0.030

#### Refinement

*R*[*F* ^2^ > 2σ(*F* ^2^)] = 0.042
*wR*(*F* ^2^) = 0.107
*S* = 1.04
2875 reflections
205 parameters
3 restraints
H atoms treated by a mixture of independent and constrained refinement
Δρ_max_ = 0.20 e Å^−3^
Δρ_min_ = −0.17 e Å^−3^

### 

Data collection: *SMART* (Bruker, 1999 ▶); cell refinement: *SAINT* (Bruker, 1999 ▶); data reduction: *SAINT*; program(s) used to solve structure: *SHELXTL* (Sheldrick, 2008) ▶; program(s) used to refine structure: *SHELXTL* ▶; molecular graphics: *SHELXTL* ▶; software used to prepare material for publication: *SHELXTL* ▶.

## Supplementary Material

Crystal structure: contains datablocks global, I. DOI: 10.1107/S1600536810051986/xu5117sup1.cif

Structure factors: contains datablocks I. DOI: 10.1107/S1600536810051986/xu5117Isup2.hkl

Additional supplementary materials:  crystallographic information; 3D view; checkCIF report

## Figures and Tables

**Table 1:** Hydrogen-bond geometry (Å, °)

*D*—H⋯*A*	*D*—H	H⋯*A*	*D*⋯*A*	*D*—H⋯*A*
N2—H1*N*⋯O1^i^	0.81 (2)	2.17 (2)	2.972 (2)	171 (2)
N2—H2*N*⋯O1	0.81 (1)	2.17 (2)	2.777 (2)	132 (2)

Symmetry code: (i) 
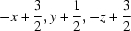
.

**Fig. 1.**
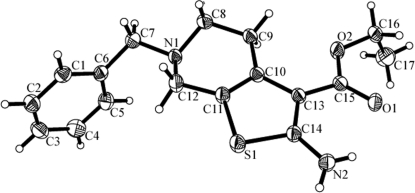
Molecular structure of the title compound. Displacement ellipsoids are drawn at the 30% probabilitylevel.

**Fig. 2.**
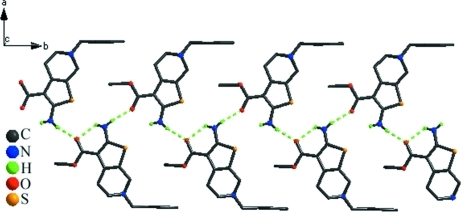
View of the one-dimensional supra-molecular chain of the title compound formed by hydrogen bonding (dashed lines). H atoms of C omitted for clarity.

## supplementary crystallographic information

### Comment

As part of an investigation of the thiophene and it's derivatives systems due to their diverse biological activities (Kidwai *et al.*, 2003; Amr *et al.*, 2006; Sherif *et al.*, 1996), we present here the crystal structure of the title compound, (I).

In the crystal structure of title compound (Fig.1), all bond lengths and bond angles have standard dimensions.

The fragments (C8 to C12) of piperidine nearly planar (mean deviation from plane within 0.0632 (1) Å) while the the six-membered piperidine ring exhibits half-chair conformation. The amino group are hydrogen bonded to the carbonyl O atom of another molecule (Table 1), forming a one-dimensional supramolecular structure (Fig. 2). In addition, there are intramolecular N—H···O hydrogen-bonding interactions in the crystal.

### Experimental

To the solution containing the ethyl 2-cyanoacetate (10 mmol, 1.06 ml), 1-benzylpiperidin-4-one (10 mmol, 1.80 ml) and powdered sulfur (12 mmol, 0.38 g) in DMF (6 ml), was under stirring triethylamine (1.20 ml) dropwise added. When the reaction was finished (TLC monitoring) the mixture was filtered with charcoal and poured into crushed ice. The formed crystals were filtered off and washed with water. The products were crystallized from ethanol.

### Refinement

All H atoms bound to C atoms were positioned geometrically and refined as riding, with C—H = 0.93 Å (CH), C—H = 0.97 Å (CH_2_) and *U*_iso_(H) = 1.2*U*_eq_(C), C—H = 0.96 Å (CH_3_) and *U*_iso_(H) = 1.5*U*_eq_(C). The H atoms bound to N atoms were located in a difference Fourier map and refined with *U*_iso_(H) = 1.2*U*_eq_(N). The N—H distances were restrained.

### Crystal data

**Table:**

C_17_H_20_N_2_O_2_S	*F*(000) = 672
*M**_r_* = 316.41	*D*_x_ = 1.294 Mg m^−^^3^
Monoclinic, *P*2_1_/*n*	Mo *K*α radiation, λ = 0.71073 Å
Hall symbol: -P 2yn	Cell parameters from 2875 reflections
*a* = 12.197 (3) Å	θ = 2.2–25.1°
*b* = 9.936 (3) Å	µ = 0.21 mm^−^^1^
*c* = 13.775 (4) Å	*T* = 293 K
β = 103.430 (4)°	Block, yellow
*V* = 1623.8 (8) Å^3^	0.25 × 0.19 × 0.14 mm
*Z* = 4	

### Data collection

**Table:**

Bruker SMART APEX CCD diffractometer	2875 independent reflections
Radiation source: fine-focus sealed tube	2122 reflections with *I* > 2σ(*I*)
graphite	*R*_int_ = 0.030
ω scans	θ_max_ = 25.1°, θ_min_ = 2.0°
Absorption correction: multi-scan (*SADABS*; Bruker, 1999)	*h* = −14→14
*T*_min_ = 0.953, *T*_max_ = 0.977	*k* = −10→11
8867 measured reflections	*l* = −16→14

### Refinement

**Table:**

Refinement on *F*^2^	Primary atom site location: structure-invariant direct methods
Least-squares matrix: full	Secondary atom site location: difference Fourier map
*R*[*F*^2^ > 2σ(*F*^2^)] = 0.042	Hydrogen site location: inferred from neighbouring sites
*wR*(*F*^2^) = 0.107	H atoms treated by a mixture of independent and constrained refinement
*S* = 1.04	*w* = 1/[σ^2^(*F*_o_^2^) + (0.0533*P*)^2^ + 0.2035*P*] where *P* = (*F*_o_^2^ + 2*F*_c_^2^)/3
2875 reflections	(Δ/σ)_max_ < 0.001
205 parameters	Δρ_max_ = 0.20 e Å^−^^3^
3 restraints	Δρ_min_ = −0.17 e Å^−^^3^

### Special details

**Table:**

Geometry. All e.s.d.'s (except the e.s.d. in the dihedral angle between two l.s. planes) are estimated using the full covariance matrix. The cell e.s.d.'s are taken into account individually in the estimation of e.s.d.'s in distances, angles and torsion angles; correlations between e.s.d.'s in cell parameters are only used when they are defined by crystal symmetry. An approximate (isotropic) treatment of cell e.s.d.'s is used for estimating e.s.d.'s involving l.s. planes.
Refinement. Refinement of *F*^2^ against ALL reflections. The weighted *R*-factor *wR* and goodness of fit *S* are based on *F*^2^, conventional *R*-factors *R* are based on *F*, with *F* set to zero for negative *F*^2^. The threshold expression of *F*^2^ > σ(*F*^2^) is used only for calculating *R*-factors(gt) *etc*. and is not relevant to the choice of reflections for refinement. *R*-factors based on *F*^2^ are statistically about twice as large as those based on *F*, and *R*- factors based on ALL data will be even larger.

### Fractional atomic coordinates and isotropic or equivalent isotropic displacement parameters (Å^2^)

**Table:**

	*x*	*y*	*z*	*U*_iso_*/*U*_eq_	
C1	0.15299 (16)	0.6448 (2)	0.47716 (17)	0.0480 (5)	
H1	0.1593	0.6354	0.4115	0.058*	
C2	0.14619 (18)	0.7720 (2)	0.51559 (19)	0.0583 (6)	
H2	0.1477	0.8472	0.4757	0.070*	
C3	0.13735 (19)	0.7877 (2)	0.6113 (2)	0.0610 (6)	
H3	0.1325	0.8734	0.6370	0.073*	
C4	0.1356 (2)	0.6766 (3)	0.67014 (19)	0.0630 (6)	
H4	0.1300	0.6873	0.7359	0.076*	
C5	0.14210 (18)	0.5487 (2)	0.63200 (17)	0.0539 (6)	
H5	0.1408	0.4740	0.6724	0.065*	
C6	0.15054 (15)	0.5310 (2)	0.53451 (16)	0.0416 (5)	
C7	0.15136 (17)	0.3930 (2)	0.48984 (17)	0.0503 (6)	
H7A	0.0800	0.3494	0.4889	0.060*	
H7B	0.1577	0.4022	0.4212	0.060*	
C8	0.23336 (17)	0.17339 (19)	0.49528 (17)	0.0480 (5)	
H8A	0.2476	0.1822	0.4292	0.058*	
H8B	0.1571	0.1401	0.4878	0.058*	
C9	0.31573 (15)	0.07232 (19)	0.55515 (16)	0.0441 (5)	
H9A	0.2875	0.0412	0.6115	0.053*	
H9B	0.3216	−0.0049	0.5136	0.053*	
C10	0.43028 (15)	0.13375 (19)	0.59228 (14)	0.0372 (5)	
C11	0.44382 (15)	0.26693 (19)	0.58452 (15)	0.0409 (5)	
C12	0.35245 (15)	0.36571 (19)	0.54262 (17)	0.0471 (5)	
H12A	0.3633	0.4473	0.5824	0.057*	
H12B	0.3552	0.3890	0.4748	0.057*	
C13	0.53359 (15)	0.06514 (18)	0.64255 (14)	0.0371 (4)	
C14	0.62303 (15)	0.15380 (19)	0.66948 (15)	0.0405 (5)	
C15	0.54785 (16)	−0.07575 (19)	0.66775 (14)	0.0400 (5)	
C16	0.45439 (19)	−0.28970 (19)	0.65815 (19)	0.0562 (6)	
H16A	0.5239	−0.3251	0.6456	0.067*	
H16B	0.3921	−0.3307	0.6106	0.067*	
C17	0.4472 (2)	−0.3267 (2)	0.7607 (2)	0.0746 (8)	
H17A	0.4497	−0.4229	0.7676	0.112*	
H17B	0.3777	−0.2935	0.7730	0.112*	
H17C	0.5095	−0.2877	0.8080	0.112*	
O1	0.63729 (11)	−0.12830 (13)	0.70893 (11)	0.0503 (4)	
O2	0.45118 (11)	−0.14512 (13)	0.64303 (12)	0.0540 (4)	
S1	0.58197 (4)	0.31782 (5)	0.63602 (5)	0.0502 (2)	
N1	0.24303 (12)	0.30618 (15)	0.54336 (13)	0.0417 (4)	
N2	0.73186 (14)	0.12651 (18)	0.71338 (16)	0.0544 (5)	
H1N	0.7738 (18)	0.1883 (17)	0.7340 (17)	0.065*	
H2N	0.7441 (19)	0.0488 (15)	0.7300 (17)	0.065*	

### Atomic displacement parameters (Å^2^)

**Table:**

	*U*^11^	*U*^22^	*U*^33^	*U*^12^	*U*^13^	*U*^23^
C1	0.0413 (11)	0.0530 (14)	0.0484 (13)	−0.0033 (10)	0.0078 (10)	0.0019 (10)
C2	0.0566 (14)	0.0454 (14)	0.0690 (17)	−0.0080 (11)	0.0066 (12)	0.0068 (12)
C3	0.0595 (15)	0.0463 (14)	0.0756 (19)	−0.0028 (11)	0.0125 (13)	−0.0119 (12)
C4	0.0689 (16)	0.0696 (17)	0.0550 (15)	0.0006 (13)	0.0232 (12)	−0.0077 (13)
C5	0.0573 (14)	0.0506 (14)	0.0553 (15)	0.0017 (11)	0.0157 (11)	0.0094 (11)
C6	0.0290 (10)	0.0431 (12)	0.0510 (13)	0.0035 (8)	0.0058 (9)	0.0013 (10)
C7	0.0397 (11)	0.0476 (13)	0.0586 (14)	0.0062 (10)	0.0012 (10)	−0.0040 (10)
C8	0.0384 (11)	0.0411 (12)	0.0595 (14)	−0.0027 (9)	0.0009 (10)	−0.0071 (10)
C9	0.0378 (11)	0.0329 (11)	0.0592 (14)	−0.0022 (8)	0.0063 (10)	−0.0038 (9)
C10	0.0346 (10)	0.0327 (10)	0.0445 (12)	−0.0012 (8)	0.0100 (9)	−0.0014 (8)
C11	0.0332 (10)	0.0334 (11)	0.0561 (13)	−0.0011 (8)	0.0100 (9)	0.0031 (9)
C12	0.0373 (11)	0.0359 (11)	0.0667 (15)	0.0017 (9)	0.0090 (10)	0.0065 (10)
C13	0.0356 (10)	0.0296 (10)	0.0459 (12)	−0.0007 (8)	0.0090 (9)	−0.0015 (8)
C14	0.0355 (10)	0.0358 (11)	0.0495 (13)	0.0015 (8)	0.0087 (9)	0.0003 (9)
C15	0.0387 (11)	0.0353 (11)	0.0456 (12)	−0.0004 (9)	0.0088 (9)	−0.0041 (9)
C16	0.0553 (14)	0.0261 (11)	0.0791 (18)	−0.0048 (9)	−0.0010 (12)	−0.0014 (10)
C17	0.0791 (18)	0.0546 (15)	0.083 (2)	−0.0141 (13)	0.0051 (15)	0.0115 (14)
O1	0.0399 (8)	0.0375 (8)	0.0689 (10)	0.0062 (6)	0.0032 (7)	0.0037 (7)
O2	0.0413 (8)	0.0291 (8)	0.0841 (11)	−0.0039 (6)	−0.0006 (7)	0.0050 (7)
S1	0.0357 (3)	0.0324 (3)	0.0794 (4)	−0.0055 (2)	0.0068 (3)	0.0047 (3)
N1	0.0319 (8)	0.0328 (9)	0.0576 (11)	0.0020 (7)	0.0048 (8)	−0.0008 (8)
N2	0.0363 (10)	0.0392 (10)	0.0810 (15)	−0.0036 (8)	−0.0002 (9)	0.0014 (10)

### Geometric parameters (Å, °)

**Table:**

C1—C2	1.380 (3)	C10—C11	1.341 (3)
C1—C6	1.383 (3)	C10—C13	1.458 (3)
C1—H1	0.9300	C11—C12	1.497 (3)
C2—C3	1.357 (3)	C11—S1	1.7445 (19)
C2—H2	0.9300	C12—N1	1.462 (2)
C3—C4	1.372 (3)	C12—H12A	0.9700
C3—H3	0.9300	C12—H12B	0.9700
C4—C5	1.384 (3)	C13—C14	1.384 (3)
C4—H4	0.9300	C13—C15	1.443 (3)
C5—C6	1.382 (3)	C14—N2	1.352 (2)
C5—H5	0.9300	C14—S1	1.736 (2)
C6—C7	1.505 (3)	C15—O1	1.224 (2)
C7—N1	1.468 (2)	C15—O2	1.340 (2)
C7—H7A	0.9700	C16—O2	1.451 (2)
C7—H7B	0.9700	C16—C17	1.482 (3)
C8—N1	1.469 (2)	C16—H16A	0.9700
C8—C9	1.521 (3)	C16—H16B	0.9700
C8—H8A	0.9700	C17—H17A	0.9600
C8—H8B	0.9700	C17—H17B	0.9600
C9—C10	1.501 (3)	C17—H17C	0.9600
C9—H9A	0.9700	N2—H1N	0.807 (15)
C9—H9B	0.9700	N2—H2N	0.809 (14)
			
C2—C1—C6	121.2 (2)	C10—C11—C12	125.70 (17)
C2—C1—H1	119.4	C10—C11—S1	112.26 (14)
C6—C1—H1	119.4	C12—C11—S1	121.95 (14)
C3—C2—C1	120.2 (2)	N1—C12—C11	109.34 (16)
C3—C2—H2	119.9	N1—C12—H12A	109.8
C1—C2—H2	119.9	C11—C12—H12A	109.8
C2—C3—C4	119.8 (2)	N1—C12—H12B	109.8
C2—C3—H3	120.1	C11—C12—H12B	109.8
C4—C3—H3	120.1	H12A—C12—H12B	108.3
C3—C4—C5	120.3 (2)	C14—C13—C15	120.63 (17)
C3—C4—H4	119.9	C14—C13—C10	111.72 (17)
C5—C4—H4	119.9	C15—C13—C10	127.61 (17)
C6—C5—C4	120.6 (2)	N2—C14—C13	128.54 (18)
C6—C5—H5	119.7	N2—C14—S1	119.99 (15)
C4—C5—H5	119.7	C13—C14—S1	111.45 (14)
C5—C6—C1	117.87 (19)	O1—C15—O2	122.38 (18)
C5—C6—C7	121.5 (2)	O1—C15—C13	124.76 (18)
C1—C6—C7	120.6 (2)	O2—C15—C13	112.84 (16)
N1—C7—C6	114.01 (16)	O2—C16—C17	112.14 (19)
N1—C7—H7A	108.8	O2—C16—H16A	109.2
C6—C7—H7A	108.8	C17—C16—H16A	109.2
N1—C7—H7B	108.8	O2—C16—H16B	109.2
C6—C7—H7B	108.8	C17—C16—H16B	109.2
H7A—C7—H7B	107.6	H16A—C16—H16B	107.9
N1—C8—C9	112.02 (16)	C16—C17—H17A	109.5
N1—C8—H8A	109.2	C16—C17—H17B	109.5
C9—C8—H8A	109.2	H17A—C17—H17B	109.5
N1—C8—H8B	109.2	C16—C17—H17C	109.5
C9—C8—H8B	109.2	H17A—C17—H17C	109.5
H8A—C8—H8B	107.9	H17B—C17—H17C	109.5
C10—C9—C8	111.19 (16)	C15—O2—C16	118.70 (15)
C10—C9—H9A	109.4	C14—S1—C11	91.59 (9)
C8—C9—H9A	109.4	C12—N1—C7	110.42 (15)
C10—C9—H9B	109.4	C12—N1—C8	109.76 (16)
C8—C9—H9B	109.4	C7—N1—C8	109.22 (15)
H9A—C9—H9B	108.0	C14—N2—H1N	118.7 (16)
C11—C10—C13	112.98 (16)	C14—N2—H2N	114.6 (16)
C11—C10—C9	119.74 (17)	H1N—N2—H2N	124 (2)
C13—C10—C9	127.21 (17)		
			
C6—C1—C2—C3	0.3 (3)	C9—C10—C13—C15	−0.3 (3)
C1—C2—C3—C4	0.2 (4)	C15—C13—C14—N2	5.0 (3)
C2—C3—C4—C5	−0.4 (4)	C10—C13—C14—N2	−177.1 (2)
C3—C4—C5—C6	0.1 (4)	C15—C13—C14—S1	−176.97 (15)
C4—C5—C6—C1	0.4 (3)	C10—C13—C14—S1	0.9 (2)
C4—C5—C6—C7	−176.62 (19)	C14—C13—C15—O1	−3.6 (3)
C2—C1—C6—C5	−0.6 (3)	C10—C13—C15—O1	178.95 (19)
C2—C1—C6—C7	176.49 (19)	C14—C13—C15—O2	175.16 (18)
C5—C6—C7—N1	−58.6 (3)	C10—C13—C15—O2	−2.3 (3)
C1—C6—C7—N1	124.5 (2)	O1—C15—O2—C16	−5.2 (3)
N1—C8—C9—C10	−43.7 (2)	C13—C15—O2—C16	176.01 (18)
C8—C9—C10—C11	10.4 (3)	C17—C16—O2—C15	86.3 (2)
C8—C9—C10—C13	−172.76 (19)	N2—C14—S1—C11	177.73 (18)
C13—C10—C11—C12	−175.99 (19)	C13—C14—S1—C11	−0.45 (17)
C9—C10—C11—C12	1.2 (3)	C10—C11—S1—C14	−0.11 (17)
C13—C10—C11—S1	0.6 (2)	C12—C11—S1—C14	176.65 (18)
C9—C10—C11—S1	177.86 (15)	C11—C12—N1—C7	−171.98 (17)
C10—C11—C12—N1	19.4 (3)	C11—C12—N1—C8	−51.5 (2)
S1—C11—C12—N1	−156.87 (15)	C6—C7—N1—C12	−61.0 (2)
C11—C10—C13—C14	−1.0 (3)	C6—C7—N1—C8	178.23 (18)
C9—C10—C13—C14	−177.97 (19)	C9—C8—N1—C12	66.7 (2)
C11—C10—C13—C15	176.68 (19)	C9—C8—N1—C7	−172.09 (18)

### Hydrogen-bond geometry (Å, °)

**Table:**

*D*—H···*A*	*D*—H	H···*A*	*D*···*A*	*D*—H···*A*
N2—H1N···O1^i^	0.81 (2)	2.17 (2)	2.972 (2)	171 (2)
N2—H2N···O1	0.81 (1)	2.17 (2)	2.777 (2)	132 (2)

Symmetry codes: (i) −*x*+3/2, *y*+1/2, −*z*+3/2.
